# Maximising the acceptability of extended time intervals between screens in the NHS Cervical Screening Programme: An online experimental study

**DOI:** 10.1177/0969141320970591

**Published:** 2020-11-11

**Authors:** Emily Hill, Martin Nemec, Laura Marlow, Susan Mary Sherman, Jo Waller

**Affiliations:** 1 Department of Behavioural Science and Health, Institute of Epidemiology and Health Care, UCL, London, UK; 2 Cancer Prevention Group, School of Cancer and Pharmaceutical Sciences, King’s College London, London, UK; 3 School of Psychology, Keele University, Keele, UK

**Keywords:** Cancer communication, human papillomavirus primary screening, information provision, cervical cancer, human papillomavirus, acceptability

## Abstract

**Objective:**

The NHS Cervical Screening Programme plans to increase the screening interval from 3 to 5 years for women aged 25–49 who test negative for human papillomavirus (HPV). This exploratory cross-sectional online survey tested the impact of different levels of information about the proposed change on acceptability of a longer interval.

**Methods:**

Women aged 18–45 (n = 585) were individually randomised to one of three information exposure groups differing in the level of information provided about the screening interval change: (1) basic information; (2) basic information with additional detail about timeline of HPV infection; (3) as (2) but with the addition of a diagram. Acceptability of the change (*favourable* and *unfavourable attitudes*) was assessed post-exposure alongside HPV timeline beliefs. We used ANOVA and regression analyses to test for between-group differences.

**Results:**

Women in Group 3 had higher scores on the *favourable attitudes* sub-scale compared with Group 1. Women in Groups 2 and 3 had more accurate timeline beliefs than those in Group 1. There were no between-group differences in *unfavourable attitudes*. After adjusting for demographic factors, a higher *favourable attitudes* score was independently predicted by being in Group 3 compared to Group 1, more accurate HPV timeline beliefs, and previous irregular or non-attendance at screening.

**Conclusions:**

Overall, acceptability of an increased screening interval was moderate, but providing women with information about the safety and rationale for this change may improve acceptability. In particular, communicating the long timeline from HPV exposure to cervical cancer may reassure women about the safety of the proposed changes.

## Introduction

Human papillomavirus (HPV) based cervical screening has recently been implemented in the UK and elsewhere,^1–3^ leading to increased interest in extending screening intervals. The superior negative predicative value of an HPV test (compared with cytology) means screening intervals can be extended with little increased risk to women.^1,4^ There are currently plans to increase the screening interval in England for women aged 25–49 from 3 to 5 years.^3^

Acceptability is an important consideration ahead of policy changes. It has been defined as ‘a multi-faceted construct which reflects the extent to which people delivering or receiving a healthcare intervention consider it to be appropriate, based on anticipated or experienced cognitive and emotional responses’^5^ (p.1). The experience of other countries suggests longer screening intervals may not be acceptable to all women, although acceptability has usually been assessed using behavioural intentions.^6–8^ In the United States, where annual screening was recommended until 2012, a survey found that only 68% of women aged 36–62 were willing to have screening every 3 years if recommended by their doctor, falling to 25% if this was 5-yearly.^8^ Likewise, a mixed-methods Canadian study^7^ explored women’s intentions to attend HPV primary screening every 4 years instead of every 2. Although 84% of women expressed intention to attend HPV-based screening 2-yearly, when the HPV test was coupled with an extended 4-yearly interval, this dropped to 54%. Ogilvie et al.’s qualitative analysis further highlighted that many women feared being screened less often.^9^ The relatively low acceptability in these studies may be partly due to the information women were given about the rationale for the screening interval extension. In Silver et al.,^8^ women were provided with minimal information, and while Ogilvie et al.^9^ informed women about the rationale of the change, this information lacked a specific statement about safety. Women may also be less accepting of extended intervals if they perceive they have a choice between more and less frequent screening.

Research in Australia following the introduction of HPV testing with extended intervals showed that some women felt their health was being endangered and devalued. Women felt an increased interval might be the result of budget cuts, and that the change could ultimately lead to missed or advanced-stage diagnoses of cervical cancer.^6^ Negative reactions may have stemmed partly from a lack of understanding regarding the changes to screening^6^; and some women in other studies have suggested their fears could be alleviated with more information about HPV testing and the interval change.^9^ There is a clear need to develop effective communication about the safety and rationale of such changes,^6,8,9^ in line with the informed choice approach to screening invitations.

The common-sense model of self-regulation of health and illness (CSM)^10^ proposes that in response to a health threat individuals form illness representations.^11,12^ These relate to the individual’s beliefs about the consequences of the health threat/illness (its effect on their life), timeline (e.g. does the illness last a long or short time; does it come and go), cause (reason for the illness), control (whether the illness can be influenced or treated) and identity (symptoms and illness label).^11,13^ Illness representations then influence the individual’s appraisal, coping outcomes and consequently their health-related decisions.^12^ In the context of longer screening intervals, we hypothesised that an understanding of the timeline from HPV acquisition to the development of cervical abnormalities and cancer would be a key element of women’s cognitive representations.

Such understanding could be enhanced by using visual aids such as infographics or diagrams. In their systematic review, Garcia-Retamero and Cokely^14^ reported that presenting health-risk information in a visual format improved risk literacy compared to providing the same health-risk information using only numbers and text. It has also been suggested that such visual aids can enhance decision making and promote healthy behaviours.^14,15^

Using the CSM, Sekhon et al.^16^ recently proposed a theoretical framework of acceptability (TFA) of healthcare interventions with seven underlying components, including feelings about the intervention (affective attitude), whether the intervention is in line with one’s values (ethicality), how likely an intervention is to accomplish its purpose (perceived effectiveness), and understanding of the intervention (intervention coherence). This approach to considering acceptability is broader than that used in previous studies, which have either used atheoretical items to gauge women’s views,^8^ or social cognition models to predict behavioural intentions on the basis of attitudes, norms and perceived behavioural control.^7,9^ Using the TFA allows for exploration of cognitive and emotional responses to the proposed change which may be independent of intention to take part.

In this study, we examined acceptability of an extended cervical screening interval among women in the target population, using the TFA as the theoretical framework. We tested the hypothesis that presenting information in a way that facilitates consistency between women’s illness representations of HPV and the accepted medical model (with regard to timeline) would result in greater acceptability of this policy change, and explored the usefulness of a diagram for improving acceptability of a prolonged screening interval.^17^

The study addressed the following research questions:

1. Does providing women with information about the long timeline between an HPV infection and cervical cancer make an increased screening interval more acceptable, and does the addition of a visual diagram enhance acceptability compared with written information alone?
2. Is acceptability of an increased screening interval influenced by women’s perceptions of the timeline between an HPV infection and cervical cancer?

## Methods

### Design

The study was a cross-sectional online survey with participants individually randomised to one of three exposure groups. The outline protocol and the full questionnaire are available on Open Science Framework (OSF) (https://osf.io/wt2a7/). The study was approved by the UCL research ethics committee (ref 15187/001). Data were collected in July 2019 while HPV primary screening was being rolled out across England.

### Participants

Women aged between 18 (approaching eligibility for screening at 25) and 45 (approaching the age when screening currently changes to 5-yearly), living in the UK, and with no personal history of cervical cancer were eligible for participation. They were recruited from an online panel hosted by Dynata Global Ltd. The panel is composed of people recruited through various channels including banner advertising displays and those who have consented to be contacted for marketing and research purposes.

As there were no previous data on which to base a sample size calculation, we powered the study to detect a small-to-medium main effect^18^ of exposure group on the primary outcome (η^2^ = 0.025, meaning that 2.5% of the variance in the primary outcome is explained by exposure group). The time and resource constraints of the study were also considered when deciding on the sample size. Assuming a 5% two-tailed significance level, 90% power and 10% attrition rate, a total of 555 participants was required to give an analysable sample of n = 166 per group.

### Materials and measures

We used an online questionnaire hosted by SurveyMonkey. Women were asked to read basic information on cervical screening including HPV primary testing taken from Public Health England (PHE) screening invitation materials, and brief details of plans to lengthen the screening interval. Women were randomised to one of three information exposure groups (see Box 1).

Box 1. Information exposure

**Table:** 

Exposure 1 (Basic): This included basic information on the change from cytology to HPV-based screening (which was being rolled out across England when the survey was carried out), extension of the screening interval from 3 to 5 years for women aged 25–49, and general information about HPV and what HPV testing involves.
Exposure 2 (Extended): This contained all the Exposure 1 content with an additional explanation of why a longer screening interval is safe: that the time it takes an HPV infection to develop into cancer is at least 10 years and that the HPV test has higher accuracy than cytology-based screening for detecting abnormalities.
Exposure 3 (Extended plus diagram): This version contained all Exposure 1 and Exposure 2 content, with the addition of a diagram illustrating the ‘HPV infection to cancer’ timeline and the difference in accuracy between the HPV and cytology tests.

#### Primary outcome

The primary outcome was acceptability of an increased screening interval. As there is not yet a validated measure of the TFA constructs, we used nine unvalidated items developed for the study (see Supplementary material).^16^ These covered concerns raised by women in previous studies of extended intervals (e.g. safety and accuracy)^6,8,9^ and four aspects of the TFA judged to be most relevant to the change (affective attitude, intervention coherence, perceived effectiveness and ethicality). Items were rated on a Likert scale ranging from 1 (‘strongly disagree’) to 5 (‘strongly agree’). Once drafted, the items were piloted with a convenience sample of eight women to check for clarity and ease of completion.

#### Secondary outcome and sociodemographic characteristics

Two items were used to assess women’s timeline beliefs regarding the progression of an HPV infection to cervical cancer. These were adapted from the ‘acute/chronic timeline’ construct of the Illness Perception Questionnaire-Revised (IPQ-R).^13^ Responses used a 5-point Likert scale ranging from 1 (‘strongly agree’) to 5 (‘strongly disagree’). See Supplementary material for survey items.

Simple items were used to collect sociodemographic data (age, ethnicity, education level, country of residence), cervical screening history, intention to attend cervical screening when next invited and previous awareness of HPV.

Education level was recoded into ‘High-level’ when participants reported having a degree or higher, ‘Mid-level’ if they achieved A-levels or equivalent, were still studying or selected ‘other’, and ‘Low-level’ if they had not achieved A-levels or equivalent. ‘Don’t know’ responses (n = 1) were excluded. Ethnic group was assessed using the 2011 UK Census question^19^ offering 14 predefined response categories. These were later recoded into ‘Any white background’ and ‘Minority ethnic background’. Overall, 14% of the sample were from a minority ethnic background in line with the 2011 UK Census.^19^

Self-reported screening attendance was assessed using three items, recoded as: ‘first timer’ (for women who had been invited and attended once), ‘regular attender’ (invited more than once and attended every time), ‘irregular attender’ (invited more than once but sometimes missed or delayed), ‘non-attender’ (invited but never attended) and ‘never invited’ (if they had not received a screening invitation).

### Procedure

Participants received an email invitation from Dynata containing a web hyper-link to the survey. Those providing consent went on to answer eligibility questions. Eligible participants were asked about their cervical screening history and intention to attend when next invited. SurveyMonkey then randomised participants to the three exposure groups (individually, with a ratio of 1:1:1). A comprehension check was carried out to ensure all participants understood that the screening interval may increase to 5 years. If this was answered incorrectly, participants were asked to re-read the information and complete the comprehension check question again. Items assessing acceptability, HPV timeline beliefs and sociodemographic characteristics were answered last.

### Statistical analyses

Analyses were exploratory and were conducted using IBM SPSS Statistics version 25.0 following a pre-registered analysis plan (https://osf.io/wt2a7/). A one-way ANOVA was performed for each acceptability and timeline item to assess whether there were between-group differences as a function of information exposure. A Bonferroni correction was used to account for the risk of Type 1 error, and a *p*-value of 0.005 was used for these analyses. For ease of interpretation, we present proportions agreeing/strongly agreeing rather than mean scores, alongside *p*-values for the associated chi-square tests. The planned ANOVA analyses are available on OSF.

The two timeline items were strongly correlated (r = 0.73) so these were combined into a single measure, with higher scores indicating more accurate (longer) timeline beliefs. An exploratory principal factor analysis was performed with the nine acceptability items; the results showed they consisted of two constructs. The first factor (*favourable attitudes*) included ethicality, two affective attitude items (‘pleased’ and ‘relieved’), perceived effectiveness and one screening coherence item (‘clear understanding’). The second factor, *unfavourable attitudes*, included three items: two affective attitude items (‘anger’ and ‘disappointment’) and one coherence item (‘confusion’). Details of the item loadings are provided in the Supplementary material. Both sub-scales showed high internal reliability with Cronbach’s alpha >0.85. All three scales were standardised to a range of 1–5. ANOVAs were used to examine between-group differences for each scale.

As there were significant between-group differences in scores on the *favourable attitudes* scale, multiple linear regression was conducted, controlling for demographic factors and screening history. In a second model, we added timeline belief score to see whether this explained additional variance.

## Results

### Sample characteristics

A total of 686 participants followed the link to the survey of whom 679 consented. Of these, 631 met the inclusion criteria and were randomised, with 11 excluded due to missing data on key variables. We examined the distribution of time taken to complete the survey for the remaining sample of 620 and removed six outliers who took more than an hour. Mean time for the remaining n = 614 was 5.16 min (SD: 5.27). We excluded those whose time was more than 2 standard deviations over the mean (over 16 min; n = 16) and those who took less than 2 min (n = 13), as these participants were unlikely to have read the information properly. This left a sample for analysis of 585 in exposure groups 1 (n = 185), 2 (n = 193) and 3 (n = 207). Demographic characteristics of each exposure group are shown in Table 1. Most women were eligible for screening, but 9% (n = 52) were aged 18–24 and so would not yet have been invited. *Favourable attitudes* about an increased cervical screening interval were moderate overall (mean = 3.42, SD = 0.80, possible range 1–5), while *unfavourable attitudes* were just below the mid-point of the scale (mean = 2.93, SD = 1.08, possible range: 1–5).

**Table 1.** Baseline characteristics of the sample (n = 585).

	Exposure group		
	Basic(n = 185)	Extended (n = 193)	Extended + diagram (n = 207)
Age in years (mean; SD)	33.4 (7.1)	34.7 (6.4)	35.0 (7.0)
Age group			
18–24 years	20 (10.8)	13 (6.7)	19 (9.2)
25–34 years	76 (41.1)	79 (40.9)	67 (32.4)
35–49 years	85 (45.9)	101 (52.3)	118 (57.0)
Country of residence			
England	155 (83.8)	173 (89.6)	176 (85.0)
Wales	11 (5.9)	4 (2.1)	13 (6.3)
Scotland	12 (6.5)	10 (5.2)	15 (7.2)
Northern Ireland	7 (3.8)	6 (3.1)	3 (1.4)
Educational level			
Low-level	67 (36.2)	62 (32.1)	75 (36.2)
Mid-level	42 (22.7)	40 (20.7)	49 (23.7)
High-level	74 (40.0)	91 (47.2)	82 (39.6)
Ethnic background			
Any White	154 (83.2)	167 (86.5)	183 (88.4)
Mixed/multiple	6 (3.2)	4 (2.1)	2 (1.0)
Asian/Asian British	14 (7.6)	13 (6.7)	9 (4.3)
Black/Black British	6 (3.2)	6 (3.1)	8 (3.9)
Arab	0 (0.0)	0 (0.0)	1 (0.5)
Other	2 (1.1)	1 (0.5)	2 (1.0)
Prefer not to say	1 (0.5)	2 (1.0)	1 (0.5)
Self-reported screening attendance			
First timer	14 (7.6)	17 (8.8)	15 (7.2)
Regular attender	81 (43.8)	97 (50.3)	96 (46.4)
Irregular attender	42 (22.7)	51 (26.4)	47 (22.7)
Non-attender	27 (14.6)	19 (8.8)	28 (13.5)
Never invited	21 (11.4)	9 (4.7)	21 (10.1)
Intention to take part in screening in future (yes)	157 (84.9)	170 (88.1)	178 (86.0)
Heard of HPV before today	148 (80.0)	145 (75.1)	164 (79.2)

Numbers in some columns do not add up to 100% due to missing data.

HPV: human papillomavirus.

### Between-group differences in acceptability

In general, participants in Group 3 (Extended plus diagram) and to a lesser extent those in Group 2 (Extended) regarded longer intervals as more acceptable than those in Group 1 (Basic) (see Table 2 for the percentage of each group agreeing or strongly agreeing with each item). As shown in Table 2, exposure group had a statistically significant effect on one of the *favourable attitude* items (‘I have a clear understanding of why the time interval is likely to increase’), with higher scores for women in Groups 2 and 3 than Group 1. There were no differences for the three individual *unfavourable attitude* items.

**Table 2.** Comparison of responses to individual acceptability items and timeline beliefs by information exposure group.

	n (%) answering agree/strongly agree			p-values for chi-square tests (df = 2)
Measures/Items	Group 1 (Basic)	Group 2(Extended)	Group 3(Extended plus diagram)	
Favourable attitudes				
I trust that the interval would be changed for the right reasons	109 (59.2)	113 (58.5)	138 (67.0)	0.16
I am confident that having a longer time interval is safe	61 (33.0)	85 (44.0)	96 (46.4)	0.02
I would feel pleased to be invited for cervical screening every 5 years	83 (44.9)	97 (50.3)	108 (52.2)	0.33
I would feel relieved to be invited for cervical screening every 5 years	68 (36.8)	85 (44.0)	99 (47.8)	0.08^ ^
I have a clear understanding of why the time interval is likely to increase	108 (58.4)	154 (79.8)	151 (72.9)	<0.001^a,b^
I believe that the HPV test is better at picking up abnormal cell changes	80 (43.2)	97 (50.3)	123 (59.4)	0.006^a^
Unfavourable attitudes				
I would feel angry if I could only have cervical screening every 5 years	64 (34.6)	54 (28.0)	71 (34.3)	0.29
I would feel disappointed if I could only have cervical screening every 5 years	64 (34.8)	73 (37.8)	87 (42.2)	0.31
The change to longer time intervals does not make any sense to me	66 (35.7)	60 (31.1)	71 (34.3)	0.62
	n (%) answering disagree/strongly disagree			
Timeline beliefs				
HPV only takes a short time to develop into cervical cancer	29 (15.8)	95 (49.2)	88 (42.7)	<0.001^a,b^
I believe an HPV infection can develop into cervical cancer very quickly	17 (9.2)	73 (37.8)	74 (35.7)	<0.001^a,b^

Wording of some items has been abbreviated. See full questionnaire on OSF for exact wording.

Bonferroni correction (calculated as alpha at 0.05 by number of comparisons, here 11) suggested a significance level of 0.005.

^a^Group 3 scores significantly higher than Group 1 at *p* < 0.05.

^b^Group 2 scores significantly higher than Group 1 at *p* < 0.05.

HPV: human papillomavirus; OSF: Open Science Framework.

We ran one-way ANOVAs exploring the effects of exposure group on the composite scales (see Table 3). There were statistically significant differences between exposure groups for *favourable attitudes* (*p* = 0.003), with post-hoc tests revealing that those in Group 3 had statistically significantly higher *favourable attitude* scores than those in Group 1. The effect size was similar to that anticipated in our power calculation (η^2^ = 0.02), and the observed power for this analysis was 87% (with an alpha of 0.05). *Unfavourable attitudes* did not vary between the three exposure groups.

**Table 3.** Between-group differences in response to each composite scale score: favourable attitudes, unfavourable attitudes and timeline beliefs.

	Mean (SD)			*p*-values for ANOVAs comparing mean scores by group
Measures (all ranges: 1–5)	Group 1 (Basic)	Group 2(Extended)	Group 3(Extended plus diagram)	
Favourable attitudes (six items)	3.26 (0.78)	3.45 (0.80)	3.53 (0.79)	0.003^a^
Unfavourable attitudes (three items)	2.99 (0.96)	2.81 (1.13)	2.99 (1.12)	0.16
Timeline beliefs (two items)	2.64 (0.77)	3.17 (1.03)	3.03 (1.08)	<0.001^a,b^

^a^Group 3 scores significantly higher than Group 1.

^b^Group 2 scores significantly higher than Group 1.

### Between-group differences in timeline beliefs

Exposure group also had a statistically significant effect on *timeline beliefs* with Groups 2 and 3 scoring higher (more accurately reflecting the long interval between HPV acquisition and cervical cancer development) than Group 1 on both items (Table 2). Overall *timeline beliefs* score was also statistically significantly higher in Groups 2 and 3 compared with Group 1 (Table 3).

### Multivariate models of favourable attitudes to changing intervals

When exposure group and screening history were entered into a multiple linear regression model of *favourable attitudes* (Table 4; Model 1), controlling for age, ethnic group and education level, the model was statistically significant [F(12, 572) = 4.11, *p* < 0.001] and predicted 7.9% of the variance. Being in Group 3 (β = 0.16, *p* = 0.001) or Group 2 (β = 0.11, *p* = 0.021) compared with Group 1 was associated with statistically significantly higher *favourable attitudes* score. *Favourable attitudes* were also statistically significantly higher among women who were self-reported irregular screening attenders (β = 0.11, *p* = 0.012), first-time attenders (β = 0.09, *p* = 0.047), non-attenders (β = 0.15, *p* = 0.001), or had not been invited for screening (β = 0.17, *p* = 0.004) compared with women who reported attending regularly.

**Table 4.** Multiple regression for predictors of score on the favourable attitudes* scale (n = 580).

	Model 1					Model 2				
		Confidence interval					Confidence interval			
	B	Lower	Upper	β	*p*	B	Lower	Upper	β	*p*
Exposure group:										
Group 1 (Basic)	Reference					Reference				
Group 2 (Extended)	0.19	0.03	0.35	0.11	0.021	0.12	–0.04	0.28	0.07	0.14
Group 3 (Extended plus diagram)	0.26	0.10	0.41	0.16	0.001	0.21	0.06	0.37	0.13	0.008
Self-reported screening attendance:										
Regular attenders	Reference					Reference				
Irregular attenders*	0.20	0.05	0.36	0.11	0.012	0.19	0.03	0.34	0.10	0.021
First-time attenders	0.26	0.04	0.51	0.09	0.047	0.29	0.04	0.54	0.10	0.025
Non-attenders	0.36	0.16	0.57	0.15	0.001	0.33	0.13	0.54	0.14	0.001
Never invited*	0.49	0.16	0.82	0.17	0.004	0.48	0.16	0.81	0.17	0.004
Timeline beliefs	–	–	–	–	–	0.14	0.07	0.20	0.17	<0.001

*See Table 3 for mean score by group.

Both models were adjusted for age, ethnic group and education level.

When *timeline beliefs* were added to the model (Table 4; Model 2), they were statistically significantly associated with *favourable attitudes* (β = .17, *p* < 0.001). The overall model remained statistically significant [F(13, 571) = 5.14, *p* < 0.001], explaining an additional 2.6% of the variance (10.5% overall). The effect of exposure group was attenuated so that Group 2 was no longer statistically significantly different from Group 1, suggesting that *timeline beliefs* were playing a mediating role in the difference in *favourable attitudes* between Groups 1 and 2. Scores remained statistically significantly higher in Group 3, with β reduced very slightly from 0.16 in Model 1 to 0.13 in Model 2. Beta values for the self-reported screening attendance variable remained largely unaffected by the addition of timeline beliefs to the model.

## Discussion

This is the first study in the UK to explore acceptability of increased cervical screening intervals for women taking part in HPV primary screening. In line with our theory-based hypotheses, we found that providing women with information about the long timeline from HPV infection to cancer development was associated with more accurate timeline beliefs and more favourable attitudes towards the proposed interval change.

Women who read additional information about HPV timeline and test accuracy had a better understanding of the reasons for the interval increase, believed HPV testing to be more effective and had a more accurate understanding of the long time needed for an HPV infection to develop into cervical cancer. Previous studies have shown that explaining the long-time interval between HPV and cervical cancer can benefit women’s understanding of the disease and influence their behavioural intentions.^20^

In addition, women who saw a diagram depicting the progression from HPV infection to abnormal cell changes and cancer, illustrating the long time needed for cancer to develop, were more likely to perceive the interval extension as acceptable. This finding is in line with reviews on the usefulness of visual aids in healthcare interventions,^14,15^ which suggest visual aids are beneficial in enhancing understanding of health information, decision making and healthy behaviours.^14,15^ Our study suggests that acceptability is another construct relevant to healthcare that may be improved by using visual aids.

We used items designed to probe four independent components of acceptability relevant to changes being proposed in screening policy. Since the items were designed for this survey, we took a data-driven approach to analysis, treating these as two independent subscales in line with our exploratory factor analysis: *favourable attitudes* and *unfavourable attitudes*. Interestingly, while information exposure had an impact on favourable attitudes, unfavourable attitudes (feelings of anger, disappointment and confusion) did not differ between the three groups. This suggests that simply providing additional information on the safety and rationale for an extended screening interval will not be sufficient to mitigate adverse emotional responses to the change. The CSM suggests that emotional processes interact with cognitive processes, but these may also act in parallel.^13,21^ As such, different interventions may be appropriate to target cognitive responses (e.g. problem-solving interventions) and emotional responses (emotion regulation interventions, e.g. management of distress).^22^ Our results are in line with this conceptualisation and suggest that some women may not benefit from cognition-based interventions alone.^22^ Different types of interventions, in addition to information provision, should be considered to address women’s emotional responses to the interval change.

Compared with regular screening attenders, women who were irregular attenders, first-time attenders, non-attenders or had never been invited for screening had more favourable attitudes towards the interval change. This difference was strongest in the non-attender and the never invited groups. It may be that those who are less engaged with the current screening programme perceive fewer problems with extended intervals since they do not have a strong feeling of being entitled to 3-yearly screening.^23,24^ Some of these women may find screening aversive or may find it difficult to find time to attend (hence attending irregularly or not at all) and may therefore welcome less frequent screening. It would be useful to explore this further in future studies.

We used acceptability rather than intention to be screened as the primary outcome, allowing us to identify concerns even among women who might intend to take part. Although acceptability can predict intention,^5^ the relationship between acceptability and behaviour is not straightforward: some women might continue to take part in less frequent screening and some may make more effort to attend because of concerns about safety if they ‘miss out’ on a screen offered every 5 years instead of every 3. For others, the change could undermine the perceived importance of screening or confidence in the screening programme, potentially leading to lower uptake. It is also possible that some women may seek additional screening through private healthcare if intervals in the NHS programme are extended. Assessing acceptability of healthcare interventions is in line with person-centred approaches to healthcare, with patient beliefs, expectations, preferences and values being taken into account,^25,26^ and more work is needed to better understand how acceptability is related to screening uptake once intervals are extended.

There are several limitations to our study. Women were asked to report on acceptability of a hypothetical change to screening, which could affect the validity of the findings. While our items assessing acceptability were selected to represent several aspects of acceptability based on theory, the scales were unvalidated so their validity and reliability are uncertain. In addition, we did not assess some components of the TFA (‘burden’, ‘opportunity costs’, ‘self-efficacy’) as these were less obviously relevant to the proposed interval change.^16^ Less screening might be expected to reduce burden and opportunity costs (not increase them), while self-efficacy for attending might not be expected to change. With hindsight, it would have been preferable to include all the TFA constructs in the measure (e.g. less frequent screening could have opportunity costs by reducing regular access to a nurse with whom to discuss other health concerns). Further development and validation of an acceptability measure would be useful. Such a measure could be used on a larger scale to obtain feedback on screening programmes. The analyses were also exploratory in nature and tested the usefulness of the TFA in the context of the screening interval change. As the change is rolled out, further work is needed to confirm the findings and to explore the impact of different communication approaches on women’s understanding and uptake of screening.

Due to the nature of the participant recruitment, information on response rate was not available and it is unclear how representative the sample was of the wider screening population. The proportions of the sample with degree- and A-level education were in line with national data,^27^ as was the proportion from ethnic minority backgrounds.^28^ Women in the pre-screening age group (18–24) were under-represented in the study. However, the aim of the study was to make between-group comparisons rather than to draw generalisable conclusions about acceptability across the population. We did not assess the impact of HPV vaccine status on acceptability of extended intervals. This would be important to explore in future research as it is possible that lower risk perceptions in vaccinated women may make less frequent screening more acceptable.

## Conclusions

Since it began in 1988, the NHS Cervical Screening Programme has been successful at reducing the incidence of cervical malignancy.^29,30^ For this trend to continue, uptake of screening is essential, and acceptability has an important part to play in this.^5^ The findings of the research demonstrate that women will need to be provided with further information before implementation of an increased screening interval occurs. Visual aids, as well as information regarding the timeline of an HPV infection and the superior accuracy of the test, appear particularly useful and should be strongly considered for inclusion in the cervical screening information materials. Different approaches will also be needed to address the negative emotional responses some women have to the idea of longer screening intervals.

## Supplemental Material

sj-pdf-1-msc-10.1177_0969141320970591 - Supplemental material for Maximising the acceptability of extended time intervals between screens in the NHS Cervical Screening Programme: An online experimental study Click here for additional data file. Supplemental material, sj-pdf-1-msc-10.1177_0969141320970591 for Maximising the acceptability of extended time intervals between screens in the NHS Cervical Screening Programme: An online experimental study by Emily Hill, Martin Nemec, Laura Marlow, Susan Mary Sherman and Jo Waller in Journal of Medical Screening

## References

[1] Aitken CA van Agt HME Siebers AG, et al. Introduction of primary screening using high-risk HPV DNA detection in the Dutch cervical cancer screening programme: a population-based cohort study. BMC Med 2019; 17: 228–233. 31829241 10.1186/s12916-019-1460-0 PMC6907114

[2] Australian Government Department of Health. National cervical screening program, https://www.woncaeurope.org/page/definition-of-general-practice-family-medicine (2017, accessed 15 May 2020).

[3] PHE. Significant landmark as primary HPV screening is offered across England, https://phescreening.blog.gov.uk/2020/01/23/significant-landmark-as-primary-hpv-screening-is-offered-across-england/ (2020, accessed 19 March 2020).

[4] Koliopoulos G Nyaga VN Santesso N, et al. Cytology versus HPV testing for cervical cancer screening in the general population. Cochrane Database Syst Rev 2017; 8: CD008587–/11. DOI: 10.1002/14651858.CD008587.pub2. 10.1002/14651858.CD008587.pub2 PMC6483676 28796882

[5] Sekhon M Cartwright M Francis JJ. Acceptability of healthcare interventions: an overview of reviews and development of a theoretical framework. BMC Health Serv Res 2017; 17: 88. 28126032 10.1186/s12913-017-2031-8 PMC5267473

[6] Obermair HM Dodd RH Bonner C, et al. ‘It has saved thousands of lives, so why change it?' Content analysis of objections to cervical screening programme changes in Australia. BMJ Open 2018; 8: e019171. 10.1136/bmjopen-2017-019171 PMC5829885 29440214

[7] Ogilvie GS Smith LW van Niekerk DJ, et al. Women's intentions to receive cervical cancer screening with primary human papillomavirus testing. Int J Cancer 2013; 133: 2934–2943. 23754203 10.1002/ijc.28324 PMC4515309

[8] Silver MI Rositch AF Burke AE, et al. Patient concerns about human papillomavirus testing and 5-year intervals in routine cervical cancer screening. Obstet Gynecol 2015; 125: 317–329. 25568994 10.1097/AOG.0000000000000638 PMC4304949

[9] Ogilvie GS Smith LW van Niekerk D, et al. Correlates of women's intentions to be screened for human papillomavirus for cervical cancer screening with an extended interval. BMC Public Health 2016; 16: 213. 26935960 10.1186/s12889-016-2865-8 PMC4776398

[10] Leventhal H Brissette I Leventhal EA. The common-sense model of self-regulation of health and illness. In: Cameron LD Leventhal H (ed.) The self-regulation of health and illness behaviour. New York: Routledge, 2003, pp.42–65.

[11] Cameron LD. Screening for cancer: illness perceptions and illness worry. In: KJ Petrie JA Weinman (ed.) Perceptions of health and illness: current research and applications. Amsterdam: Harwood Academic Publishers, 1997, pp.291–322.

[12] Hagger M Orbell S. A confirmatory factor analysis of the revised Illness Perception Questionnaire (IPQ-R) in a cervical screening context. Psychol Health 2005; 20: 161–173.

[13] Moss-Morris R Weinman J Petrie K, et al. The Revised Illness Perception Questionnaire (IPQ-R). Psychol Health 2002; 17: 1–16.

[14] Garcia-Retamero R Cokely ET. Designing visual aids that promote risk literacy: a systematic review of health research and evidence-based design heuristics. Hum Factors 2017; 59: 582–627. 28192674 10.1177/0018720817690634

[15] McCrorie A Donnelly C McGlade K. Infographics: healthcare communication for the digital age. Ulster Med J 2016; 85: 71–75. 27601757 PMC4920488

[16] Sekhon M Cartwright M Francis JJ. Acceptability of health care interventions: a theoretical framework and proposed research agenda. Br J Health Psychol 2018; 23: 519–531. 29453791 10.1111/bjhp.12295

[17] Marlow LAV Wardle J Waller J, et al. Human papillomavirus (HPV) information needs: a theoretical framework. J Fam Plann Reprod Health Care 2009; 35: 29–33. 19126314 10.1783/147118909787072432 PMC3970721

[18] Cohen J. Statistical power analysis for the behavioral sciences. 2nd ed. Hillsdale: Lawrence Erlbaum, 1988.

[19] Office for National Statistics 2011 Census, https://www.ons.gov.uk/ons/guide-method/census/2011/the-2011-census/2011-census-questionnaire-content/2011-census-questions---england.pdf (accessed 18 May 2020).

[20] Marlow LAV Ryan M Waller J. Increasing the perceived relevance of cervical screening in older women who do not plan to attend screening. Sex Transm Infect 2020; 96: 20–25. 31395750 10.1136/sextrans-2019-054120 PMC7029243

[21] Hagger M Orbell S. A revised common sense model of illness self-regulation. Epub ahead of print 31 March 2020. DOI: 10.31234/osf.io/hxqnd. 10.1080/17437199.2021.1878050 33461402

[22] Cameron LD Jago L. Emotion regulation interventions: a common-sense model approach. Br J Health Psychol 2008; 13: 215–221. 18302809 10.1348/135910708X288800

[23] Kahneman D Knetsch JL Thaler RH. Anomalies: the endowment effect, loss aversion, and status quo bias. J Econ Perspect 1991; 5: 193–206.

[24] Severens JL Brunenberg DEM Fenwick EAL, et al. Cost-effectiveness acceptability curves and a reluctance to lose. PharmacoEconomics 2005; 23: 1207–1214. 16336015 10.2165/00019053-200523120-00005

[25] Van Royen P Beyer M Chevallier P, et al. The research agenda for general practice/family medicine and primary health care in Europe. Part 3. Results: person centred care, comprehensive and holistic approach. Eur J Gen Pract 2010; 16: 113–119. 20438283 10.3109/13814788.2010.481018

[26] WONCA Europe. The European definition of General Practice/Family Medicine: Short version, https://www.woncaeurope.org/page/definition-of-general-practice-family-medicine (2005, accessed 6 May 2020).

[27] Graduates in the UK labour market: 2017, https://www.ons.gov.uk/employmentandlabourmarket/peopleinwork/employmentandemployeetypes/articles/graduatesintheuklabourmarket/2017#steady-increase-in-the-number-of-graduates-in-the-uk-over-the-past-decade (accessed 21 September 2020).

[28] Ethnicity facts and figures. Population of England and Wales, https://www.ethnicity-facts-figures.service.gov.uk/uk-population-by-ethnicity/national-and-regional-populations/population-of-england-and-wales/latest (accessed 21 September 2020).

[29] Landy R Pesola F Castañón A, et al. Impact of cervical screening on cervical cancer mortality: estimation using stage-specific results from a nested case-control study. Br J Cancer 2016; 115: 1140–1146. 27632376 10.1038/bjc.2016.290 PMC5117785

[30] Cancer Research UK. Cervical cancer statistics, https://www.cancerresearchuk.org/health-professional/cancer-statistics/statistics-by-cancer-type/cervical-cancer#heading-Zero (accessed 19 May 2020).

