# Supplementary material for: Maximising the acceptability of extended time intervals between screens in the NHS Cervical Screening Programme: An online experimental study
Source: J Med Screen. 2020 Nov 11;28(3):333–40. doi: 10.1177/0969141320970591 (PMC8366181; doi:10.1177/0969141320970591)
Supplement: sj-pdf-1-msc-10.1177_0969141320970591 - Supplemental material for Maximising the acceptability of extended time intervals between screens in the NHS Cervical Screening Programme: An online experimental study [file sj-pdf-1-msc-10.1177_0969141320970591.pdf]

Maximising the acceptability of extended time intervals between screens in the NHS Cervical Screening Programme: an online experimental study

Supplementary material

1. Development of acceptability items

2. Survey items (pages 2-8)

3. Factor loadings from the exploratory factor analysis of acceptability items (page 9)

1. Development of acceptability items

Item wording	Theoretical Framework of Acceptabilty construct
‘I am confident that having a longer time interval between cervical screens is safe’	Ethicality
‘I trust that the interval between cervical screens would be changed for the right reasons’	Ethicality
‘I would feel pleased to be invited for cervical screening every 5 years instead of every 3 years’	Affective attitude (positive)
‘I would feel relieved to be invited for cervical screening every 5 years instead of every 3 years’	Affective attitude (positive)
‘I would feel angry if I could only have cervical screening every 5 years instead of every 3 years’	Affective attitude (negative)
‘I would feel disappointed if I could only have cervical screening every 5 years instead of every 3 years’	Affective attitude (negative)
‘I have a clear understanding of why the time interval between screens is likely to increase’	Intervention coherence
‘The change to longer time intervals between screens doesn’t make any sense to me’	Intervention coherence
‘I believe that the HPV test is better than the current (cytology) test at picking up abnormal cell changes’	Perceived effectiveness

2. Survey items

Firstly, we'd like to ask you a few questions to check that you're a good fit for our study.

About you

*What is your gender?

- ❑ Female
- ❑ **Male (exclude)**
- ❑ **Prefer not to say (exclude)**

*Where do you currently live?

- ❑ England
- ❑ Wales
- ❑ Scotland
- ❑ Northern Ireland
- ❑ **Other (Exclude)**

*How old are you?

- ❑ 18-30
- ❑ 31-45
- ❑ **46 or over (Exclude)**

*Have you ever been diagnosed with cervical cancer?

- ❑ **Yes (Exclude)**
- ❑ No
- ❑ Prefer not to say (Exclude)

Thank you for filling in the initial questions. We'd now like to introduce you to the main part of the questionnaire. This survey is about cervical screening, also known as the 'smear' or 'Pap' test. In the UK, the test is regularly offered to women aged 25 to 64 years. Women receive an invitation in the post and can make an appointment to have screening at their GP surgery or sometimes at a local sexual health or well-woman clinic.

*Have you ever been for cervical screening (sometimes called a 'smear' or 'Pap' test)?

- ❑ Yes
- ❑ No, I have been invited but have never had cervical screening
- ❑ No, I have never been invited to have cervical screening
- ❑ I do not know what cervical screening is
- ❑ Prefer not to say

*Which of these statements best describes your cervical screening history?

- ❑ I have attended cervical screening once
- ❑ I have attended cervical screening more than once

* When invited, how regularly have you been for cervical screening?

- ❑ I have been for cervical screening every time I have been invited
- ❑ I have sometimes delayed or missed going for cervical screening
- ❑ Unsure

*Do you plan to go for cervical screening when you are next invited?

- ❑ Yes, definitely
- ❑ Yes, probably
- ❑ Probably not
- ❑ Definitely not

Please read the following information about cervical screening carefully. Once you have finished reading, press ‘next’.

Exposure 1 (Basic):

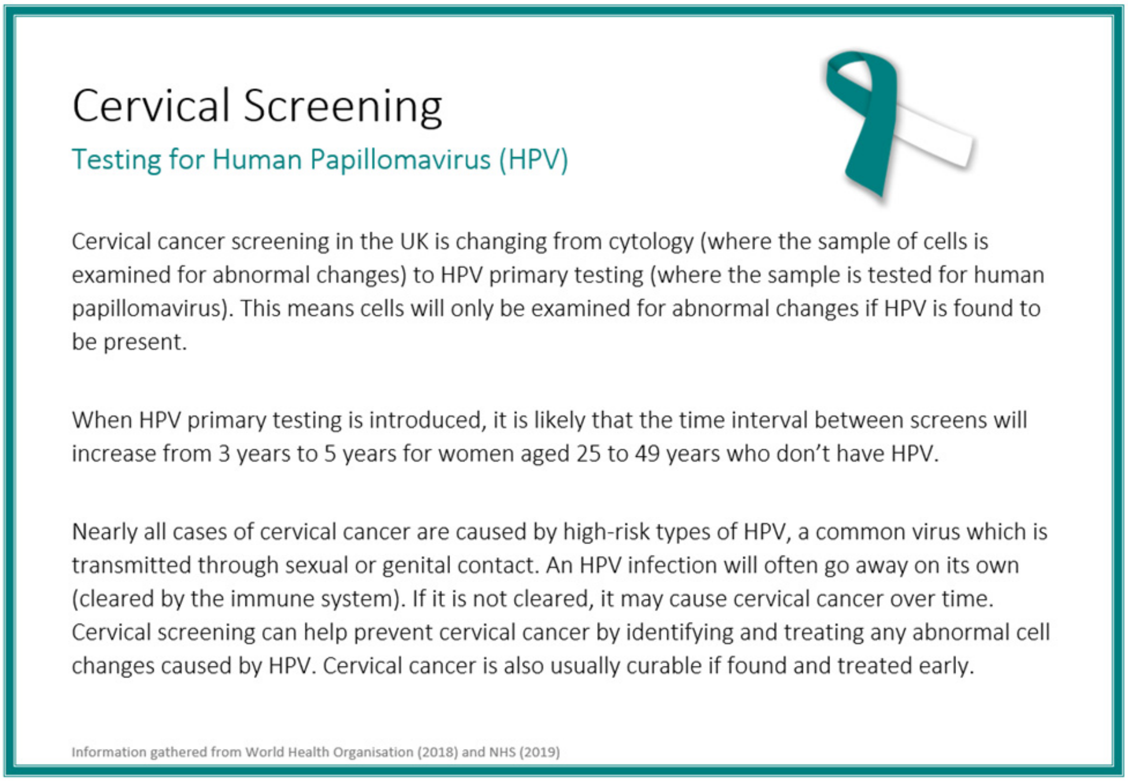

*Did you read the information about HPV?

- ❑ Yes
- ❑ No

Exposure 2 (Extended):

Cervical Cancer Screening

Testing for Human Papillomavirus (HPV)

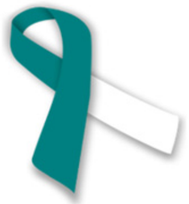

Cervical cancer screening in the UK is changing from cytology (where the sample of cells is examined for abnormal changes) to HPV primary testing (where the sample is tested for human papillomavirus). This means cells will only be examined for abnormal changes if HPV is found to be present.

When HPV primary testing is introduced, it is likely that the time interval between screens will increase from 3 years to 5 years for women aged 25 to 49 years who don't have HPV. This longer interval will be safe because it takes at least 10 years for HPV to develop into cancer. As well as this, the HPV test is better than the current test at picking up problems so there is a very low chance of the test missing something.

Nearly all cases of cervical cancer are caused by high-risk types of HPV, a common virus which is transmitted through sexual or genital contact. An HPV infection will often go away on its own (cleared by the immune system). If it is not cleared, it may cause cervical cancer over time. Cervical screening can help prevent cervical cancer by identifying and treating any abnormal cell changes caused by HPV. Cervical cancer is also usually curable if found and treated early.

Information gathered from World Health Organisation (2018) and NHS (2019)

*Did you read all 3 paragraphs about HPV?

- ❑ Yes
- ❑ No

Exposure 3 (Extended plus diagram):

Cervical Cancer Screening

Testing for Human Papillomavirus (HPV)

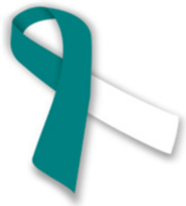

Cervical cancer screening in the UK is changing from cytology (where the sample of cells is examined for abnormal changes) to HPV primary testing (where the sample is tested for human papillomavirus). This means cells will only be examined for abnormal changes if HPV is found to be present.

When HPV primary testing is introduced, it is likely that the time interval between screens will increase from 3 years to 5 years for women aged 25 to 49 years who don't have HPV. This longer interval will be safe because it takes at least 10 years for HPV to develop into cancer. As well as this, the HPV test is better than the current test at picking up problems so there is a very low chance of the test missing something.

Nearly all cases of cervical cancer are caused by high-risk types of HPV, a common virus which is transmitted through sexual or genital contact. An HPV infection will often go away on its own (cleared by the immune system). If it is not cleared, it may cause cervical cancer over time. Cervical screening can help prevent cervical cancer by identifying and treating any abnormal cell changes caused by HPV. Cervical cancer is also usually curable if found and treated early.

Information gathered from World Health Organisation (2018) and NHS (2019)

The diagram below shows the timeline from an HPV infection to cervical cancer.

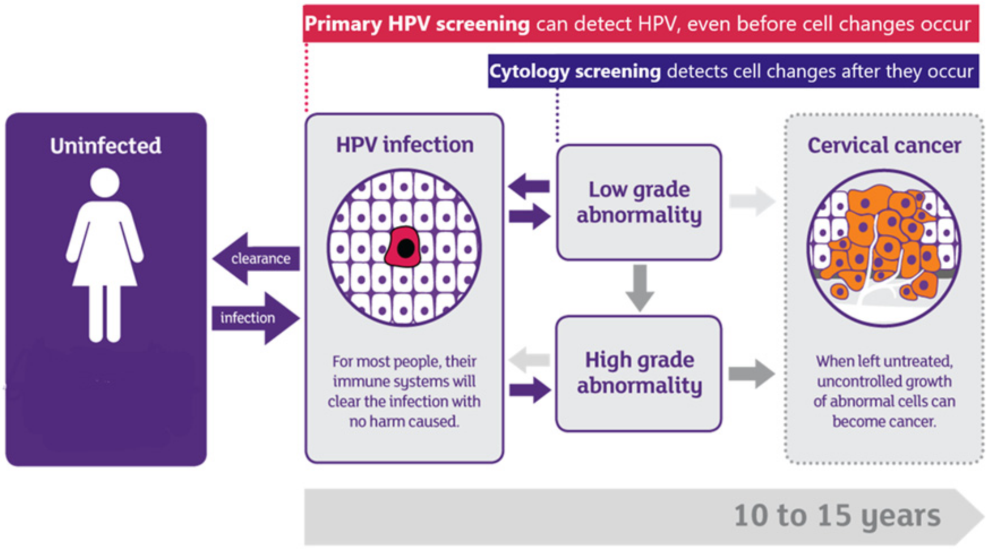

Information gathered from World Health Organisation (2018), NHS (2019), and NSW Cancer Institute (2019)

*Did you read the information and look at the diagram about HPV?

- ☐ Yes
- ☐ No

* Once HPV primary screening is introduced, the time interval between screens for women age 25-49 is likely to be:

- ❑ Every 3 years (route back to information page)
- ❑ Every 5 years
- ❑ Not sure (route back to information page)

Having read this information, please indicate how much you agree or disagree with the following statements:

*I am confident that having a longer time interval between cervical screens is safe.

- ❑ Strongly Agree
- ❑ Agree
- ❑ Unsure
- ❑ Disagree
- ❑ Strongly Disagree

*I have a clear understanding of why the time interval between screens is likely to increase.

- ❑ Strongly Agree
- ❑ Agree
- ❑ Unsure
- ❑ Disagree
- ❑ Strongly Disagree

* I would feel pleased to be invited for cervical screening every 5 years instead of every 3 years.

- ❑ Strongly Agree
- ❑ Agree
- ❑ Unsure
- ❑ Disagree
- ❑ Strongly Disagree

*The change to longer time intervals between screens doesn't make any sense to me.

- ❑ Strongly Agree
- ❑ Agree
- ❑ Unsure
- ❑ Disagree
- ❑ Strongly Disagree

*I would feel angry if I could only have cervical screening every 5 years instead of every 3 years.

- ❑ Strongly Agree
- ❑ Agree
- ❑ Unsure
- ❑ Disagree
- ❑ Strongly Disagree

* I trust that the interval between cervical screens would be changed for the right reasons.
- ❑ Strongly Agree
- ❑ Agree
- ❑ Unsure
- ❑ Disagree
- ❑ Strongly Disagree

* I would feel relieved to be invited for cervical screening every 5 years instead of every 3 years.
- ❑ Strongly Agree
- ❑ Agree
- ❑ Unsure
- ❑ Disagree
- ❑ Strongly Disagree

* I believe that the HPV test is better than the current (cytology) test at picking up abnormal cell changes
- ❑ Strongly Agree
- ❑ Agree
- ❑ Unsure
- ❑ Disagree
- ❑ Strongly Disagree

*HPV only takes a short time to develop into cervical cancer.
- ❑ Strongly Agree
- ❑ Agree
- ❑ Unsure
- ❑ Disagree
- ❑ Strongly Disagree

*I would feel disappointed if I could only have cervical screening every 5 years instead of every 3 years.
- ❑ Strongly Agree
- ❑ Agree
- ❑ Unsure
- ❑ Disagree
- ❑ Strongly Disagree

*I believe an HPV infection can develop into cervical cancer very quickly.
- ❑ Strongly Agree
- ❑ Agree
- ❑ Unsure
- ❑ Disagree
- ❑ Strongly Disagree

Finally, we'd like to ask you a few general questions about yourself.

*How old are you? Please enter your exact age below:

*Do you have any educational qualifications? If so, what is the highest qualification you have?

- ❑ Degree or higher
- ❑ ONC/BTEC/NVQ
- ❑ A levels or Highers
- ❑ O Level or GCSE equivalent
- ❑ No formal qualifications
- ❑ Still studying
- ❑ Other
- ❑ Don't know

***What is your ethnic group?**

<u>White</u>

- ❑ English / Welsh / Scottish / Northern Irish / British
- ❑ Irish
- ❑ Gypsy or Irish Traveller
- ❑ European
- ❑ Other (write in)

<u>Mixed / multiple ethnic groups</u>

- ❑ White and Black Caribbean
- ❑ White and Black African
- ❑ White and Asian
- ❑ Other (write in)

<u>Asian / Asian British</u>

- ❑ Indian
- ❑ Pakistani
- ❑ Bangladeshi
- ❑ Chinese
- ❑ Other (write in)

<u>Black / African / Caribbean / Black British</u>

- ❑ African
- ❑ Caribbean
- ❑ Other (write in)

<u>Other Ethnic group</u>

- ❑ Arab
- ❑ Other (write in)
- ❑ Prefer not to say

*Before today, had you heard of HPV?

- ❑ Yes
- ❑ No
- ❑ Don't know

3. Factor loadings from the exploratory factor analysis of acceptability items

Survey item	Factor	
	Favourable attitudes	Unfavourable attitudes
I am confident that having a longer time interval between cervical screens is safe	.81	.08
I trust that the time interval between cervical screens would be changed for the right reasons	.77	.02
I would feel pleased to be invited for cervical screening every 5 years instead of every 3 years	.73	.15
I would feel relieved to be invited for cervical screening every 5 years instead of every 3 years	.70	.12
I believe that the HPV test is better than the current (cytology) test at picking up abnormal cell changes	.63	-.24
I have a clear understanding of why the time interval between screens is likely to increase	.53	.004
I would feel angry if I could only have cervical screening every 5 years instead of every 3 years	-.13	.99
I would feel disappointed if I could only have cervical screening every 5 years instead of every 3 years	-.02	.89
The change to longer time intervals between screens doesn't make any sense to me	.07	.62

Note: Item loadings were determined by principal axis factoring with promax rotation.
